# Uma Apresentação Invulgar de Cardite de Lyme e Bloqueio Atrioventricular Sensível à Adenosina

**DOI:** 10.36660/abc.20230228

**Published:** 2024-01-29

**Authors:** André Alexandre, Diana Ribeiro, Maria João Sousa, Hipólito Reis, João Silveira, Severo Torres

**Affiliations:** 1 Centro Hospitalar Universitário de Santo António Porto Portugal Departamento de Cardiologia, Centro Hospitalar Universitário de Santo António (CHUDSA), Porto – Portugal; 2 Faculdade de Medicina e Ciências Biomédicas Universidade do Porto Porto Portugal ICBAS – Faculdade de Medicina e Ciências Biomédicas , Universidade do Porto , Porto – Portugal

**Keywords:** Doença de Lyme, Bloqueio Atrioventricular, Cardiomiopatia, Adenosina, Teofilina

## Introdução

A cardite de Lyme (CL) é uma manifestação relativamente rara do estágio inicial disseminado da doença de Lyme. ^1 , 2^ A apresentação mais frequente da CL é o bloqueio atrioventricular (AV), caracterizado por rápidas flutuações no grau e que ocorre em aproximadamente 80-90% dos pacientes. ^1 , 3^ Outras possíveis manifestações clínicas da CL são miocardite, pericardite, cardiomiopatia dilatada e insuficiência cardíaca. ^1 , 4^ O curso clínico é geralmente leve, de curta duração e, na maioria dos casos, completamente reversível após tratamento antibiótico adequado. ^1^ Na verdade, o bloqueio AV de alto grau associado à doença de Lyme normalmente remite nos primeiros dez dias de terapia antibiótica. ^3^ Os autores apresentam o caso de uma jovem com diagnóstico de doença de Lyme precocemente disseminada e manifestações cardíacas, na qual os distúrbios de condução AV persistiram após quatro semanas de tratamento antibiótico adequado e levaram a um diagnóstico alternativo.

## Apresentação do caso

Paciente do sexo feminino, 20 anos, praticante de ginástica acrobática de 12 a 18 anos, apresentou quadro de dor torácica pleurítica aguda e dispneia. À admissão apresentava-se hemodinamicamente estável, levemente taquicárdica (100/min), apirética, com ausculta pulmonar e cardíaca normais e sem sinais congestivos.

**Figura 1:**
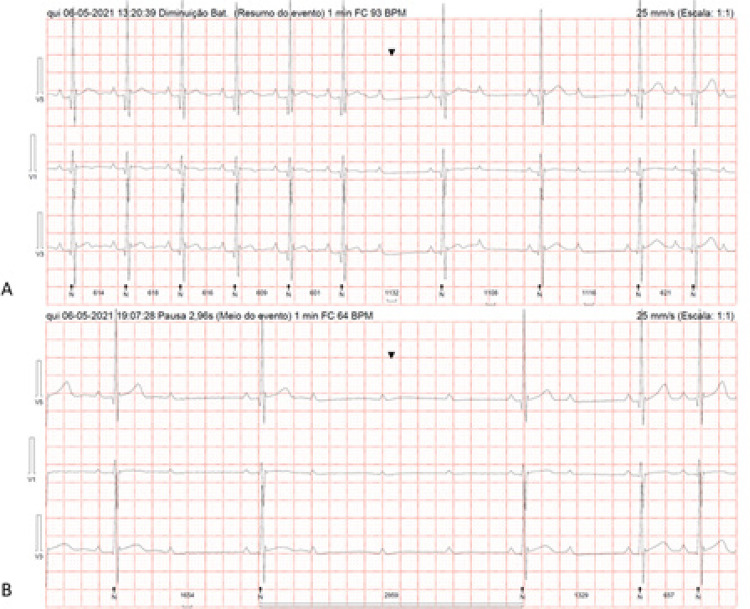
– Monitoramento Holter demonstrando dois episódios de bloqueio atrioventricular (AV) paroxístico após o término do tratamento com antibióticos. O monitoramento contínuo do Holter revelou que os distúrbios da condução AV (incluindo bloqueio AV paroxístico de alto grau) persistiram após quatro semanas de tratamento antibiótico apropriado. O painel A mostra um bloqueio AV 2:1 de segundo grau. O painel B mostra um bloqueio AV de alto grau com assistolia ventricular de 2,96 segundos.

O paciente apresentou eletrocardiograma mostrando taquicardia sinusal (102/min) e inversão da onda T nas derivações inferior e V3-V6 ( Figura 1 suplementar). A radiografia de tórax estava normal. A análise sanguínea revelou elevação dos biomarcadores cardíacos (troponina T de alta sensibilidade 0,085 ng/mL [valores de referência 0,000–0,014]) e elevação dos parâmetros inflamatórios (proteína C reativa 135 mg/L [0-5] e velocidade de hemossedimentação 104 mm [0-19]). A ecocardiografia transtorácica mostrou hipocinesia do segmento basal do septo interventricular anterior, função sistólica biventricular preservada, sem disfunção valvar, nem derrame pericárdico. Considerando a suspeita de miocardite aguda, a paciente foi internada em unidade de terapia intensiva cardíaca. Durante a internação, o monitoramento cardíaco contínuo revelou vários episódios assintomáticos de bloqueio AV paroxístico (Figura 2 suplementar), incluindo bloqueio AV Mobitz I de segundo grau, bloqueio AV 2:1 de segundo grau e bloqueio AV de alto grau (3:1 e 4). :1).

Apesar da ausência de um contexto de risco epidemiológico específico para *Borrelia spp* . (como evidências de exposição a carrapatos, lesões articulares, diarreia ou lesões cutâneas), a paciente foi procurada para realização de testes sorológicos para doença de Lyme. As recomendações atuais incluem o uso de um ensaio imunoenzimático sensível (ELISA), seguido por um ensaio de imunotransferência Western para amostras que produzam resultados positivos ou equívocos. ^5 , 6^ No nosso caso, o exame sorológico inicial com teste ELISA mostrou IgM limítrofe positivo (0,30 RU/mL [<0,20]) com teste IgG ELISA negativo. Assim, foi realizado teste Western-Blot IgM, confirmando positividade e recente infecção por *Borrelia spp* .. Além disso, testes ELISA IgM e IgG subsequentes foram realizados dois meses depois, o primeiro permanecendo claramente positivo (0,38 RU/mL [< 0,20]) e o último ainda negativo. Os resultados dos testes da doença de Lyme de primeiro e segundo níveis relatados aqui apoiam a lógica combinada de sensibilidade e especificidade do algoritmo de dois níveis, em oposição ao uso de apenas um teste. ^6^ Além disso, os testes treponêmicos e não treponêmicos (VDRL; FTA-ABS) foram negativos, excluindo sífilis. Os testes de biologia molecular para um extenso painel de vírus respiratórios produziram resultados negativos (RSV, INF-A, INF-B, Parainfluenza 1, 2, 3, 4, Adenovírus, Metapneumovírus, Bocavírus, Rinovírus, Enterovírus, Coronavírus), assim como a sorologia testes para HIV, HBV e HCV. A paciente era imune a EBV, CMV, Parvovírus, Coxsackievirus e Enterovírus (IgM negativo; IgG positivo). Os testes de cultura para *S. pyogenes* e o título de antiestreptolisina O foram negativos. Os testes imunológicos foram normais. Portanto, é improvável que tenha ocorrido reatividade cruzada no nosso caso, o que permitiu a confirmação do diagnóstico de doença de Lyme. A ressonância magnética cardiovascular foi realizada uma semana após a admissão e não apresentou alterações dignas de nota. A tomografia por emissão de pósitrons excluiu sarcoidose cardíaca.

Considerando o diagnóstico de doença de Lyme disseminada precocemente com manifestações cardíacas de bloqueio AV de alto grau, a paciente foi tratada com ceftriaxona intravenosa durante quatro semanas. A resolução completa das queixas de dor torácica e dispneia foi documentada. No entanto, na nossa paciente, o distúrbio de condução AV (incluindo bloqueio AV de alto grau) persistiu após quatro semanas de tratamento antibiótico adequado, conforme demonstrado pela monitorização Holter ( Figura 1 ). Portanto, este caso pode ser um curso extremamente raro de CL ou pode ser um distúrbio de condução AV assintomático anterior que se revelou juntamente com a doença de Lyme.

O bloqueio AV paroxístico pode ser categorizado em três tipos diferentes: intrínseco, vagal extrínseco e idiopático extrínseco (“bloqueio AV sensível à adenosina”). Portanto, esta paciente realizou uma investigação diagnóstica para posterior caracterização. O teste ergométrico mostrou condução AV normal 1:1 em frequências cardíacas elevadas, sem distúrbios de condução nem disritmias e tolerância normal ao exercício (11,1 METs). O estudo eletrofisiológico (EPS) não apresentou alterações dignas de nota, descartando bloqueio AV paroxístico intrínseco (bem como quaisquer sequelas crônicas de CL). Quanto à possibilidade de bloqueio AV paroxístico vagal extrínseco, como esses episódios ocorreram dois anos após o descondicionamento da ginástica acrobática, era menos provável que o bloqueio AV extrínseco vagal contribuísse para o bloqueio AV paroxístico. Por outro lado, considerando que o bloqueio AV paroxístico de alto grau ocorreu sem prolongamento do ciclo PP ou prolongamento do intervalo PR e sem desencadeamento por batimentos prematuros nem por variações de frequência, essas características favoreceram o diagnóstico de bloqueio AV paroxístico idiopático extrínseco (também conhecido como “ bloqueio AV sensível à adenosina”).

A paciente iniciou teofilina 400 mg duas vezes ao dia e, após uma semana de tratamento, o monitoramento Holter demonstrou redução significativa dos distúrbios de condução AV. O monitoramento Holter antes da teofilina mostrou cerca de 78 ondas P bloqueadas, incluindo bloqueio AV Mobitz I de segundo grau, bloqueio AV 2:1 de segundo grau e bloqueio AV de alto grau (3:1 e 4:1), com duas assistolias ventriculares significativas, a maior das quais foi de 2.960 milissegundos devido ao bloqueio AV de alto grau (4:1) [ Figura 1 ]. A monitorização Holter após a administração de teofilina revelou uma redução notável de aproximadamente 38% das ondas P bloqueadas (de 78 para 48), principalmente devido ao bloqueio AV Mobitz I de segundo grau, com apenas dois episódios de bloqueio AV 3:1 e sem mais assistolia ventricular mais de 2.000 milissegundos. Considerando que a paciente era assintomática e como o “bloqueio AV sensível à adenosina” não evolui para formas persistentes de bloqueio AV, ela recebeu alta sem necessidade de marca-passo permanente.

Aos 18 meses de acompanhamento, o paciente permanece em terapia com teofilina, com redução dos distúrbios de condução AV, sem queixas cardíacas e sem necessidade de marcapasso permanente.

## Discussão

Este é um caso de uma jovem com diagnóstico de doença de Lyme disseminada precocemente e manifestações cardíacas, na qual os distúrbios de condução AV persistiram após quatro semanas de tratamento antibiótico adequado, levando a um diagnóstico alternativo de bloqueio AV paroxístico idiopático extrínseco (“bloqueio AV sensível à adenosina”).

A CL é uma manifestação relativamente rara de um estágio inicial disseminado da doença de Lyme. ^1 , 2^ O teste sorológico é o principal meio de diagnóstico laboratorial da doença de Lyme. ^5^ Sabe-se que a sensibilidade dos testes de dois níveis utilizando ensaios sorológicos disponíveis comercialmente depende do estágio da infecção. ^6^ Foram observadas faixas de sensibilidade de 40 a 87% em pacientes com doença disseminada precocemente (CL), e diferenças na duração e disseminação da doença nos pacientes testados podem explicar essa variabilidade. ^6^ Além disso, algumas doenças semelhantes (como fibromialgia, mononucleose infecciosa, esclerose múltipla, artrite reumatoide e sífilis) são documentadas como causadoras de reatividade cruzada em ensaios sorológicos para a doença de Lyme. ^6^ As recomendações atuais incluem o uso de um imunoensaio enzimático sensível ou ensaio de imunofluorescência, seguido por um ensaio de imunotransferência Western para amostras que produzam resultados positivos ou equívocos. ^5^ Ensaios sorológicos que utilizam um segundo imunoensaio enzimático no lugar do ensaio imunoblot ocidental são alternativas aceitáveis, embora não sejam obrigatórios para estabelecer o diagnóstico. ^5^ No nosso caso, o exame sorológico inicial com teste ELISA mostrou IgM limítrofe positivo (0,30 RU/mL [<0,20]) com teste IgG ELISA negativo. Assim, foi realizado teste Western-Blot IgM, confirmando positividade e recente Borrelia spp. infecção. Além disso, testes ELISA IgM e IgG subsequentes foram realizados dois meses depois, o primeiro permanecendo claramente positivo (0,38 RU/mL [< 0,20]) e o último ainda negativo. Os resultados dos testes da doença de Lyme de primeiro e segundo níveis relatados aqui apoiam a lógica combinada de sensibilidade e especificidade do algoritmo de dois níveis, em oposição ao uso de apenas um teste. ^6^ Além disso, os testes treponêmicos e não treponêmicos foram negativos (excluindo sífilis), assim como todos os demais testes sorológicos e imunológicos. Portanto, é improvável que tenha ocorrido reatividade cruzada no nosso caso, o que permitiu a confirmação do diagnóstico de doença de Lyme.

A apresentação mais frequente da CL é o bloqueio AV, caracterizado por rápidas flutuações de grau. ^1 , 3^ O curso clínico é geralmente leve, de curta duração e, na maioria dos casos, completamente reversível após tratamento antibiótico adequado. ^1^ Neste caso clínico, o distúrbio de condução AV persistiu após quatro semanas de tratamento antibiótico adequado. Portanto, levantou-se a hipótese de que poderia ser uma sequela crônica da CL (sendo um curso extremamente raro da doença de Lyme), ou que esse paciente poderia ter um distúrbio de condução AV assintomático prévio que se revelou junto com a doença de Lyme. A literatura atual expõe três tipos diferentes de bloqueio AV paroxístico: intrínseco, vagal extrínseco e idiopático extrínseco. ^7^ O bloqueio AV paroxístico intrínseco é devido a uma doença intrínseca do sistema de condução AV e progride para formas persistentes de bloqueio AV. ^7^ O bloqueio AV paroxístico vagal extrínseco está ligado ao efeito do sistema nervoso parassimpático na condução cardíaca e está envolvido na “síncope reflexa”. ^7^ O bloqueio AV paroxístico idiopático extrínseco está associado a baixos níveis de adenosina endógena e supostamente está envolvido na “síncope sensível à adenosina”. Caracteriza-se por bloqueio AV paroxístico completo sem prolongamento do ciclo PP ou prolongamento do intervalo PR, não é desencadeado por batimentos prematuros nem por variações de frequência e não progride para formas persistentes de bloqueio AV. ^8^ A prevalência deste tipo de bloqueio é provavelmente subdiagnosticada devido ao fraco reconhecimento e à sua imprevisibilidade. ^7^ De acordo com as Diretrizes ESC de 2021 sobre estimulação cardíaca e terapia de ressincronização cardíaca, o diagnóstico de bloqueio AV por hipersensibilidade à adenosina é apoiado por um EPS normal, bem como pela ausência de início do bloqueio por batimentos atriais ou ventriculares prematuros, nem aumento da frequência cardíaca (bloqueio AV taqui-dependente) ou diminuição da frequência cardíaca (bloqueio AV bradi-dependente). ^8 , 9^ Como os pacientes com baixos níveis plasmáticos de adenosina são altamente suscetíveis à adenosina exógena e endógena, o tratamento com teofilina tem sido investigado e parece ser eficaz, podendo ser considerado uma alternativa ao marca-passo permanente em pacientes com síncope recorrente. ^7 , 10 , 11^

Este caso ilustra um cenário desafiador de CL com bloqueio AV de alto grau, que persistiu após tratamento antibiótico apropriado e apresentou características importantes que apoiam o diagnóstico de bloqueio AV paroxístico idiopático extrínseco (“bloqueio AV sensível à adenosina”). Assim, esta paciente iniciou teofilina, obtendo redução significativa dos distúrbios de condução AV e, posteriormente, sem necessidade de marca-passo permanente.

## Conclusão

“Bloqueio AV sensível à adenosina” é um tipo de bloqueio AV paroxístico que provavelmente é subdiagnosticado devido à sua imprevisibilidade e fraco reconhecimento. O tratamento com teofilina parece ser eficaz e pode ser considerado uma alternativa ao marca-passo permanente em pacientes de baixo risco.
