# Supplementary material for: Uma Apresentação Invulgar de Cardite de Lyme e Bloqueio Atrioventricular Sensível à Adenosina
Source: Arq Bras Cardiol. 2024 Jan 29;121(1):e20230228. [Article in Portuguese] doi: 10.36660/abc.20230228 (PMC11098578; doi:10.36660/abc.20230228)
Supplement: Translation

# Translation: An Unusual Presentation of Lyme Carditis and Adenosine-Sensitive Atrioventricular Block

**Authors:** André Alexandre, Diana Ribeiro, Maria João Sousa, Hipólito Reis, João Silveira, Severo Torres

**Affiliations:** 1 Portugal Departamento de Cardiologia, Centro Hospitalar Universitário de Santo António (CHUDSA), Porto – Portugal; 2 Portugal ICBAS – Faculdade de Medicina e Ciências Biomédicas, Universidade do Porto, Porto – Portugal

**Keywords:** Lyme Disease, Atrioventricular Block, Cardiomyopathy, Adenosine

## Introduction

Lyme carditis (LC) is a relatively rare manifestation of the early disseminated stage of LC. ^1 , 2^ The most frequent presentation of LC is atrioventricular (AV) block characterized by rapid fluctuations in the grade and that occurs in approximately 80-90% of patients. ^1 , 3^ Other possible clinical manifestations of LC are myocarditis, pericarditis, dilated cardiomyopathy, and heart failure. ^1 , 4^ The clinical course is usually mild, short-term, and in most cases completely reversible after adequate antibiotic treatment. ^1^ Indeed, high-grade AV block associated with Lyme disease typically resolves within the first ten days of antibiotic therapy. ^3^ The authors present a case of a young female diagnosed with early disseminated Lyme disease and cardiac manifestations, in whom AV conduction disturbances persisted after four weeks of appropriate antibiotic treatment and led to an alternative diagnosis.

## Case Presentation

A 20-year-old female, practitioner of acrobatic gymnastics from age 12 to 18 years, presented with acute pleuritic chest pain and shortness of breath. At admission, she was hemodynamically stable, mildly tachycardic (100/min), apyretic, with normal lung and cardiac auscultation and no congestive signs.

The patient had an electrocardiogram showing sinus tachycardia (102/min) and T-wave inversion in inferior and V3-V6 leads (Supplementary Figure 1 ). The chest radiograph was normal. Blood analysis revealed an elevation of cardiac biomarkers (high-sensitivity troponin T 0.085 ng/mL [reference values 0.000–0.014]) and an elevation of inflammatory parameters (C-reactive protein 135 mg/L [0-5] and erythrocyte sedimentation rate 104 mm [0-19]). Transthoracic echocardiography showed hypokinesis of the basal segment of the anterior interventricular septum, preserved biventricular systolic function, no valvular dysfunction, nor pericardial effusion. Considering the suspicion of acute myocarditis, the patient was admitted to an intensive cardiac care unit. During hospitalization, continuous cardiac monitoring revealed several asymptomatic episodes of paroxysmal AV block (Supplementary Figure 2), including second-degree Mobitz I AV block, second-degree 2:1 AV block, and high-grade AV block (3:1 and 4:1).

**Figure 1:** – Holter monitoring demonstrating two episodes of paroxysmal atrioventricular (AV) block after completion of antibiotic treatment. Continuous Holter monitoring revealed that AV conduction disturbances (including paroxysmal high-grade AV block) persisted after four weeks of appropriate antibiotic treatment. Panel A shows a second-degree 2:1 AV block. Panel B shows a high-grade AV block with a ventricular asystole of 2.96 seconds.

Despite the absence of a specific epidemiological risk context for *Borrelia spp.* (such as evidence of tick exposure, joint lesions, diarrhea, or skin lesions), the patient was sought for serological testing for Lyme disease. Current recommendations include using a sensitive enzyme-linked immunosorbent assay (ELISA), followed by a western immunoblot assay for specimens yielding positive or equivocal results. ^5 , 6^ In our case, initial serologic examination with ELISA test showed a borderline positive IgM (0.30 RU/mL [< 0.20]) with negative IgG ELISA test. Thus, an IgM Western-Blot test was performed, confirming positivity and recent *Borrelia spp.* infection. Also, subsequent IgM and IgG ELISA tests were performed two months later, the former remaining clearly positive (0.38 RU/mL [< 0.20]) and the latter still negative. The first- and second-tier Lyme disease testing results reported here support the combined sensitivity and specificity rationale of the two-tiered algorithm as opposed to the use of just one test. ^6^ Besides that, treponemal and nontreponemal tests (VDRL; FTA-ABS) were negative, excluding syphilis. Molecular biology tests for an extensive panel of respiratory viruses yielded negative results (RSV, INF-A, INF-B, Parainfluenza 1, 2, 3, 4, Adenovirus, Metapneumovirus, Bocavirus, Rhinovirus, Enterovirus, Coronavirus), as did the serology tests for HIV, HBV, and HCV. The patient was immune to EBV, CMV, Parvovirus, Coxsackievirus, and Enterovirus (IgM negative; IgG positive). Culture tests for *S. pyogenes* and antistreptolysin O titer were negative. Immunological tests were normal. Therefore, it is unlikely that cross-reactivity occurred in our case, which allowed the diagnosis of Lyme disease to be confirmed. Cardiovascular magnetic resonance was performed one week after admission and was unremarkable. Positron emission tomography excluded cardiac sarcoidosis.

Considering the diagnosis of early disseminated Lyme disease with cardiac manifestations of high-grade AV block, the patient was treated with intravenous ceftriaxone over four weeks. Complete resolution of chest pain and dyspnea complaints were documented. Nevertheless, in our patient, the AV conduction disturbance (including high-grade AV block) persisted after four weeks of appropriate antibiotic treatment, as demonstrated by Holter monitoring ( Figure 1 ). Therefore, this case could be an extremely rare course of LC, or it could be a prior asymptomatic AV conduction disturbance that has revealed itself along with Lyme disease.

Paroxysmal AV block can be categorized into three different types: intrinsic, extrinsic vagal, and extrinsic idiopathic (“adenosine-sensitive AV block”). Hence, this patient pursued a diagnostic workup for further characterization. The exercise stress test showed normal 1:1 AV conduction at high heart rates, no conduction disturbances nor dysrhythmias, and normal exercise tolerance (11.1 METs). The electrophysiology study (EPS) was unremarkable, ruling out intrinsic paroxysmal AV block (as well as any chronic sequelae of LC). As for the possibility of extrinsic vagal paroxysmal AV block, since these episodes occurred two years after deconditioning from acrobatic gymnastics, it was less likely that extrinsic vagal contributed to paroxysmal AV block. On the other hand, considering that paroxysmal high-grade AV block occurred without PP cycle lengthening or PR interval prolongation and without triggering by premature beats nor by rate variations, these features favored the diagnosis of an extrinsic idiopathic paroxysmal AV block (also known as “adenosine-sensitive AV block”).

The patient was started on theophylline 400 mg twice daily, and after one week of treatment, the Holter monitoring demonstrated a significant reduction in AV conduction disturbances. Holter monitoring before theophylline showed around 78 blocked P waves, including second-degree Mobitz I AV block, second-degree 2:1 AV block, and high-grade AV block (3:1 and 4:1), with two significant ventricular asystoles, the largest of which was 2960 milliseconds due to high-grade AV block (4:1) [ Figure 1 ]. Holter monitoring following theophylline administration revealed a noteworthy reduction of approximately 38% of blocked P waves (from 78 to 48), mostly due to second-degree Mobitz I AV block, with only two episodes of 3:1 AV block and no ventricular asystole longer than 2000 milliseconds. Considering the patient was asymptomatic and since “adenosine-sensitive AV block” does not progress to persistent forms of AV block, she was discharged without the need for a permanent pacemaker.

At 18 months of follow-up, the patient remains on theophylline therapy, with a reduction in AV conduction disturbances, no cardiac complaints, and no need for a permanent pacemaker.

## Discussion

This is a case of a young female diagnosed with early disseminated Lyme disease and cardiac manifestations, in whom AV conduction disturbances persisted after four weeks of appropriate antibiotic treatment, leading to an alternative diagnosis of extrinsic idiopathic paroxysmal AV block (“adenosine-sensitive AV block”).

LC is a relatively rare manifestation of an early disseminated stage of Lyme disease. ^1 , 2^ Serologic testing is the principal means of laboratory diagnosis of Lyme disease. ^5^ It is known that the sensitivity of two-tiered testing using commercially available serology-based assays is dependent on the stage of infection. ^6^ Sensitivity ranges from 40 to 87% were observed in patients with early disseminated disease (LC), and differences in disease duration and dissemination in tested patients may explain this variability. ^6^ In addition, some look-alike diseases (such as fibromyalgia, infectious mononucleosis, multiple sclerosis, rheumatoid arthritis, and syphilis) are documented as causing cross-reactivity in serologic assays for Lyme disease. ^6^ Current recommendations include using a sensitive enzyme immunoassay or immunofluorescence assay, followed by a western immunoblot assay for specimens yielding positive or equivocal results. ^5^ Serologic assays that utilize a second enzyme immunoassay in place of western immunoblot assay are acceptable alternatives, although not mandatory to establish the diagnosis. ^5^ In our case, initial serologic examination with ELISA test showed a borderline positive IgM (0.30 RU/mL [< 0.20]) with negative IgG ELISA test. Thus, an IgM Western-Blot test was performed, confirming positivity and recent Borrelia spp. infection. Also, subsequent IgM and IgG ELISA tests were performed two months later, the former remaining clearly positive (0.38 RU/mL [< 0.20]) and the latter still negative. The first- and second-tier Lyme disease testing results reported here support the combined sensitivity and specificity rationale of the two-tiered algorithm as opposed to use of just one test. ^6^ Besides that, treponemal and nontreponemal tests were negative (excluding syphilis), as were all other serological and immunological tests. Therefore, it is unlikely that cross-reactivity occurred in our case, which allowed the diagnosis of Lyme disease to be confirmed.

The most frequent presentation of LC is AV block, characterized by rapid fluctuations in the grade. ^1 , 3^ The clinical course is usually mild, short-term, and, in most cases, completely reversible after adequate antibiotic treatment. ^1^ In this clinical case, the AV conduction disturbance persisted after four weeks of appropriate antibiotic treatment. Therefore, it was hypothesized that it might be a chronic sequela of LC (being an extremely rare course of Lyme disease), or this patient might have a prior asymptomatic AV conduction disturbance that revealed itself along with Lyme disease. Current literature exposes three different types of paroxysmal AV block: intrinsic, extrinsic vagal, and extrinsic idiopathic. ^7^ Intrinsic paroxysmal AV block is due to an intrinsic disease of the AV conduction system and progresses to persistent forms of AV block. ^7^ Extrinsic vagal paroxysmal AV block is linked to the effect of the parasympathetic nervous system on cardiac conduction and is involved in “reflex syncope”. ^7^ Extrinsic idiopathic paroxysmal AV block is associated with low levels of endogenous adenosine and is supposed to be involved in “adenosine-sensitive syncope”. It is characterized by paroxysmal complete AV block without PP cycle lengthening or PR interval prolongation, is not triggered by premature beats nor by rate variations, and does not progress to persistent forms of AV block. ^8^ The prevalence of this type of block is likely underdiagnosed due to poor recognition and its unpredictability. ^7^ According to 2021 ESC Guidelines on cardiac pacing and cardiac resynchronization therapy, the diagnosis of adenosine hypersensitivity AV block is supported by an unremarkable EPS, as well as the absence of initiation of the block by atrial or ventricular premature beats, nor increased heart rate (tachy-dependent AV block) or decreased heart rate (brady-dependent AV block). ^8 , 9^ Since patients with low plasma adenosine levels are highly susceptible to exogenous and endogenous adenosine, theophylline treatment has been investigated and it seems to be effective and may be considered an alternative to permanent pacing in patients with recurrent syncope. ^7 , 10 , 11^

This case illustrates a challenging scenario of LC with high-grade AV block, which persisted after appropriate antibiotic treatment and had key features supporting the diagnosis of extrinsic idiopathic paroxysmal AV block (“adenosine-sensitive AV block”). In accordance, this patient was started on theophylline, achieving a significant reduction in AV conduction disturbances and, subsequently, no need for a permanent pacemaker.

## Conclusion

“Adenosine-sensitive AV block” is a type of paroxysmal AV block that is likely underdiagnosed due to its unpredictability and poor recognition. Theophylline treatment seems to be effective and may be considered an alternative to permanent pacing in low-risk patients.
